# New Insights into the Role of Glutathione in the Mechanism of Fever

**DOI:** 10.3390/ijms21041393

**Published:** 2020-02-19

**Authors:** Sylwia Wrotek, Justyna Sobocińska, Henryk M. Kozłowski, Małgorzata Pawlikowska, Tomasz Jędrzejewski, Artur Dzialuk

**Affiliations:** 1 Department of Immunology, Faculty of Biological and Veterinary Sciences, Nicolaus Copernicus University, 1 Lwowska Str., 87-100 Torun, Poland; j.sobocinska@umk.pl (J.S.); mikolajkozlowski@doktorant.umk.pl (H.M.K.); m_pawlikowska@umk.pl (M.P.); tomaszj@umk.pl (T.J.); 2 Department of Genetics, Faculty of Biological Sciences, Kazimierz Wielki University, 10 Powstańców Wielkopolskich Ave., 85-090 Bydgoszcz, Poland

**Keywords:** fever, inflammation, immunity, antioxidants, glutathione modulators, oxidative stress

## Abstract

Glutathione is one of the most important and potent antioxidants. The development of pharmacological compounds that can either increase or decrease glutathione concentrations has allowed investigation into the role of glutathione in various biological processes, including immune responses. Recent findings have shown that glutathione not only affects certain factors involved in immunological processes but also modifies complex immune reactions such as fever. Until recently, it was not known why some patients do not develop fever during infection. Data suggest that fever induction is associated with oxidative stress; therefore, antioxidants such as glutathione can reduce pyrexia. Surprisingly, new studies have shown that low glutathione levels can also inhibit fever. In this review, we focus on recent advances in this area, with an emphasis on the role of glutathione in immune responses accompanied by fever. We describe evidence showing that disturbed glutathione homeostasis may be responsible for the lack of fever during infections. We also discuss the biological significance of the antipyretic effects produced by pharmacological glutathione modulators.

## 1. Fever—A Brief Overview

Fever is a well-coordinated pathophysiological phenomenon associated with infection and trauma that is manifested by an increase in body temperature above normal. Previous studies have shown that the preoptic area of the hypothalamus plays an important role in maintaining a stable body temperature [1]. Currently, it is believed that the actual internal temperature of endotherms such as mammals is compared with the *set point*. If body temperature is below this reference temperature, various heat retention and heat production responses are activated. Many sickness behaviors such as chills and goose pimples, assuming the fetal position to reduce body surface area, or wearing thick clothing and seeking warmer environments can be observed. Systemic symptoms such as headache, somnolence, and a decrease in motor activity, as well as hypophagia (a decrease in food intake), which leads to loss of body mass, may also accompany fever [2,3]. When the fever signal is no longer present and the *set point* reverts back to “normal”, the body temperature returns to the physiological range by activation of heat loss mechanisms such as sweating [4,5,6]. 

Fever is a part of the response known as the acute phase reaction, consisting of a host of immunologic, endocrinologic, and neurologic alterations [7]. It is triggered by microbial factors and products known as exogenous pyrogens or pathogen associated molecular patterns (PAMPs). Structures such as lipopolysaccharides (LPS), peptidoglycans, porin complexes, lipoteichoic acid, lipoarabinomannans, bacterial DNA, mycoplasma lipoproteins, and staphylococcal and streptococcal superantigens constitute a major group of the pyrogens of gram-negative, gram-positive, and mycobacterial origin [8,9,10]. 

Although a negative attitude regarding fever can be observed in many patients, the increase in body temperature is actually an important alarm signal. It has been shown in many cases that infectious fever is beneficial for healing [11,12,13,14], but in some medical situations, e.g., stroke, the beneficial effect of fever is controversial [15]. Recently, many reviews have focused on discussing the fever-induced mechanism which boosts the effectiveness of the immune response against various infections [16,17,18,19]. Generally, researchers who investigate this issue believe that fever is an adaptive response aimed at restoring the homeostasis that was disrupted by traumatic and infectious events [12,20]. 

Fever is considered beneficial because an elevated body temperature enhances the activity of immune factors and cells. A rise in body temperature triggers an increase in bactericidal activities of neutrophils and macrophages, T cell proliferation and differentiation, B cell proliferation, and the production of antibodies or stimulation of acute-phase protein synthesis [5,11,21]. At the same time, it impairs the replication of many microorganisms [18,22,23]. Evidence from clinical studies indicates that there is an association between body temperature and survival of infected patients. Young et al. [24] concluded that reducing temperature during infection may be harmful, because fever in patients critically ill with infection is associated with a decreased risk of death. Furthermore, children with chickenpox who were treated with acetaminophen (paracetamol) were shown to take a longer time for total curing of lesions than placebo-treated controls [25]. A retrospective observation of human volunteers infected with influenza A showed a relationship between antipyretic therapy and prolonged illness [26].

Interestingly, there are clinical reports suggesting a decreased frequency of fever or even the lack of capability to generate fever within certain groups of patients. The reason for this phenomenon is not known. Fewer fevers have long been recognized amongst cancer patients. The idea that deficiency of fever in the medical history of the patient corresponds with a high risk of cancer is supported in the literature [27,28,29,30]. On the other hand, there are a number of prospective and retrospective studies indicating that febrile infections lower the risk of cancer and can be associated with spontaneous remission of various tumors [31,32]. These observations have been confirmed and have been described in recent studies [33,34]. Surprisingly, in spite of evidence for the benefits of moderate fever, the prevailing practice in the general population is to block fever by taking antipyretic drugs [35]. 

## 2. Fever Induction and Antipyresis

**Figure 1:**
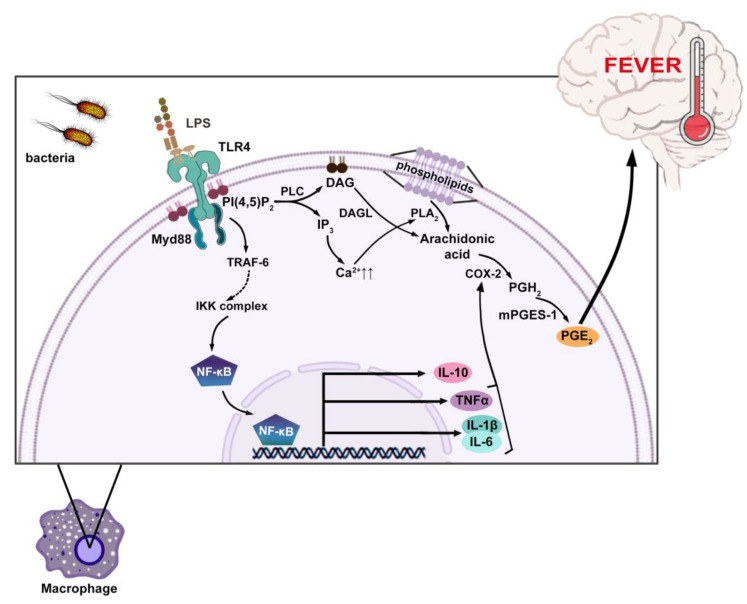
The mechanism of fever. Lipopolysaccharide (LPS) released from bacteria is recognized by Toll-like receptor 4 (TLR4). It provides signal transmission through TRAF6 and IKK family kinases to induce NF-κB-dependent gene expression, resulting in the release of pyrogenic tumor necrosis factor α (TNFα), interleukin (IL)1-β and IL-6. TLR4 activation also triggers the hydrolysis of phosphatidylinositol 4,5-bisphosphate (PI(4,5)P2) to diacylglycerol (DAG) and inositol trisphosphate (IP3) by phospholipase C (PLC). Diacylglycerol lipase (DAGL) hydrolyses diacylglycerol (DAG) presented in membrane releasing free arachidonic acid (AA). Additionally, IP3 stimulates calcium liberation from the endoplasmic reticulum to the cytoplasm, which, in turn, drives the activation of phospholipase A2 (PLA2) to release arachidonic acid from membrane phospholipids. AA is then metabolized by cyclooxygenase (COX-2) to prostaglandin H2 (PGH2). Subsequently, microsomal prostaglandin E2 synthase (mPGES-1) converts PGH2 to prostaglandin E2 (PGE2), a major mediator of fever.

In spite of attempts to describe the molecular mechanism of fever in homoeothermic organisms [12,36], it is still not fully understood. Currently, it is assumed that fever is produced after contact between circulating exogenous, pathogen-derived pyrogens and the Toll-like receptors located on host cells (Figure 1). In the laboratory, the most extensively and thoroughly studied pyrogen is lipopolysaccharide (LPS) [9,15,37,38,39]. The pyretic signal is then transmitted into the cell and activates nuclear factor κB (NF-κB). Mice without a functional NF-κB gene do not respond with fever after injection of LPS [9]. This is due to the fact that NF-κB regulates the synthesis of endogenous pyrogens, including cytokines such as interleukin (IL)-1β, IL-6 and tumor necrosis factor α (TNF-α) [40,41]. According to the current understanding of the fever induction process, these cytokines induce downstream mediators of fever, for example, triggering the liberation of arachidonic acid from membrane phospholipids, activation of cyclooxygenase (COX-2). COX-2 mediates the enzymatic conversion of arachidonic acid to prostaglandin H_2_ (PGH_2_). Further action of microsomal prostaglandin E_2_ synthase (mPGES-1) on PGH_2_ results in the production of prostaglandin E_2_ (PGE_2_), a final fever-inducing mediator of the febrile response [42,43]. Although PGE_2_ is fundamental in the febrile response, some cytokines and other inflammatory mediators may activate fever independent of PGE_2_ [44]. Other than PGE_2_, inflammatory mediators that may disrupt thermal balance include bradykinin [45], corticotropin releasing hormone [46], nitric oxide [47], macrophage inflammatory protein1 (MIP-1) [48], endothelin [49], preformed pyrogenic factors (PFPF), and substance P [6,44]. The pyrogenic signal that is generated as a result of peripheral inflammatory challenge is likely transmitted to the brain where responses can be generated to cause a rise in body temperature [43]. Importantly, factors that mediate fever are not involved in normal thermoregulation [50].

Antipyretics are substances that have a role in determining the upper limit of a fever or can inhibit fever completely. They are grouped into two categories: exogenous antipyretics and endogenous antipyretics. Exogenous antipyretics include nonsteroidal anti-inflammatory drugs (NSAIDs), acetaminophen, and corticosteroids [51]. Each exerts its effects at different points in the febrile response pathway. Acetaminophen and NSAIDs inhibit cyclooxygenase (COX)-mediated synthesis of prostaglandins from arachidonic acid [52]. Corticosteroids block the transcription of pyrogenic cytokines and inducible COX-2 via interactions involving the glucocorticoid receptor. It has been shown that aspirin and NSAIDs also have COX-independent antipyretic activity. Aspirin induces cytochrome P-450, which causes a shift in arachidonic acid metabolism toward cytochrome P-450-mediated production of antipyretic epoxyeicosanoids [52,53]. Since many NSAID-induced side effects are known, antipyretics should be used for specific indications, like other drugs, and not for fever per se [54,55,56,57].

Endogenous antipyretics include various factors such as hormones (e.g., glucocorticoids and α-melanocyte stimulating hormone), neuropeptides (e.g., arginine vasopressin) and cytokines, among others [43]. Studies have established that arginine vasopressin is released during fever and reduces fever by its action at the type I vasopressin receptor [58]. α-Melanocyte stimulating hormone reduces fever and is more than 25,000 times more potent than acetaminophen [59]. Glucocorticoids inhibit the synthesis of pyrogenic cytokines such as IL-6 and TNF-α [60]. There are also cytokines whose antipyretic properties have been established. The most investigated is IL-10, which has been shown to depress the production of TNF-α, IL-1β, and IL-6 in response to LPS [61]. Furthermore, it has been found that IL-10 deficiency or neutralization causes exacerbation of endotoxic fever [62].

## 3. Redox Status Modulates Thermal Response in Organisms 

There are data showing that fever is dependent on redox status. Riedel and Maulik [63] demonstrated the presence of lipid peroxidation products in the plasma, brain, liver, and heart during a febrile response in rats. Recently, Gomes et al. [64] found increased ROS levels in the liver, brown adipose tissue, and hypothalamus after the induction of fever in rats.

It has been found that sulfur-containing drugs, such as methylene blue and α-lipoic acid, inhibit oxygen radical production and prevent fever [63,65]. Treating rabbits with an oxygen radical scavenger such as aspirin was shown to prevent the LPS-induced rise of hydroperoxides and the inactivation of catalase, and, as a consequence, abolished fever [65]. Based on these data, it has been postulated that fever is a response to oxidative stress [63,65]. Ettebong and Nwafor [66] found that the medical plant *Eleucine indica* reduces yeast and amphetamine-induced pyrexia in a dose-dependent manner. *E. indica* is well known for its antioxidant activity since it produces high levels of antioxidant enzymes such as superoxide dismutase, catalase, and glutathione. Since reducing agents such as vitamin C, vitamin E, and carotenoids have not been found to substantially affect thermoregulation and fever, thiols have been suggested to play a crucial role in the pathways contributing to temperature homeostasis [65]. 

## 4. Glutathione 

**Figure 2:**
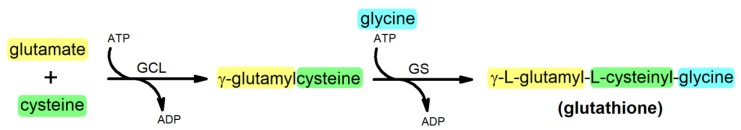
Glutathione synthesis. Three essential amino acids—glutamate, cysteine, and glycine—combine to form the tripeptide glutathione (GSH). At the beginning, cysteine is joined to glutamate through the action of glutamate cysteine ligase (GCL) to produce γ-glutamylcysteine, which proceeds to link with glycine via glutathione synthase (GS) action.

Glutathione (GSH) is the principal intracellular antioxidant buffer. It is predominately distributed in the cytosol (about 90%) and to a lesser extent in the subcellular organelles, such as the mitochondria, nucleus, and endoplasmic reticulum [67,68]. GSH is a ubiquitous, thiol-containing tripeptide produced by most mammalian cells. To synthesize glutathione, organisms require three amino acids: glutamic acid, cysteine, and glycine (Figure 2). The synthesis of GSH from its three amino acid precursors takes place in the cytosol. This process is catalyzed in all organisms by two enzymes: glutamate-cysteine ligase, regarded as the rate-limiting enzyme of the pathway, and glutathione synthase. The first step is controlled by negative feedback from its end product, GSH. When GSH is consumed and feedback inhibition is lost, the availability of cysteine as a precursor can become the limiting factor [67,69]. 

**Figure 3:**
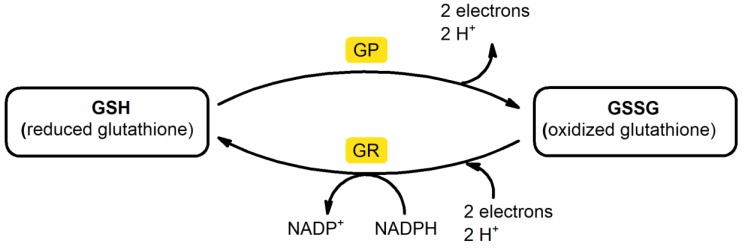
Glutathione exists in both the thiol-reduced (GSH) form and the disulfide-oxidized (GSSG) form. GSH:GSSG recycling is catalyzed by GSH peroxidase (GP) and GSH reductase (GR).

Glutathione exists in the reduced GSH form and oxidized glutathione disulfide (GSSG) form. Since GSH has a sulfhydryl group on the cysteinyl portion, it possesses a strong electron-donating character (Figure 3). As electrons are lost, the molecule becomes oxidized, and two of these molecules become linked (dimerized) by a disulfide bridge to form glutathione disulfide (GSSG). This linkage is reversible upon reduction. Although intracellular GSH mainly exists as a monomer in reduced form (more than 98% of GSH), severe oxidative stress (OS) can overcome the ability of the cell to reduce GSSG to GSH leading to accumulation of GSSG [69]. Thus, the ratio of GSH and GSSG is considered to be a marker of OS [70].

GSH is the principal intracellular antioxidant, which may act directly by scavenging reactive oxygen and nitrogen species and plays an essential role in several metabolic and cellular processes. Deficiency of GSH causes cellular risk for oxidative damage, and thus, as expected, GSH imbalance is observed in a wide range of pathological conditions including cardiometabolic and cardiovascular diseases, tuberculosis, HIV, diabetes, neurodegenerative diseases, infertility, and cancer [70,71,72,73]. Although it is widely accepted that the GSH antioxidant defenses of the body decrease linearly with age [74], studies on old rats that had never been sick before revealed GSH was at the same level as in young rats [29].

### 4.1. Glutathione Affects the Immune System and Signaling Molecules Involved in the Mechanism of Fever

The role of GSH in host defense against oxidative stress and intoxication is well established. Besides its function as an intracellular redox buffer, GSH exerts a key role in a series of immune processes. GSH is essential for some functions of both the innate and adaptive immune systems including the action of antigen presenting cells, such as dendritic cells [75], and T-lymphocyte proliferation [76,77]. The antigen presenting cells require GSH to present antigens on their major histocompatibility complex (MHC) molecules. This process starts with the degradation of antigens by the proteasome. One of the first steps in antigen degradation and processing is the reduction of disulfide bonds, which requires GSH [78,79]. The antigens then move to a more acidic environment, that facilitates further unfolding, cleavage by endopeptidases, and binding to MHC molecules [80].

Recently, a new glutathione-dependent molecular mechanism by which the human immune system activates its immune cells was discovered. T cells effectively ward off pathogens if a gene, *Gclc*, which is essential for glutathione production, is expressed within them. Mak et al. [81] discovered that after contact with pathogens, glutathione stimulates energy metabolism in T cells, so they can grow, divide, and fight off intruders. It has been shown that glutathione is an important molecular switch for the immune system. 

Furthermore, GSH affects the production of most inflammatory cytokines and is required to maintain adequate interferon (IFN)-γ production by dendritic cells [82]. Based on these data, Dröge and Breitkreutz [83] claimed that the immune system is highly affected by the level of GSH in the body. However, for many years, it was not known whether disrupted GSH levels alter a complex immune mechanism such as fever, which involves the action of many components of the immune system.

**Figure 4:**
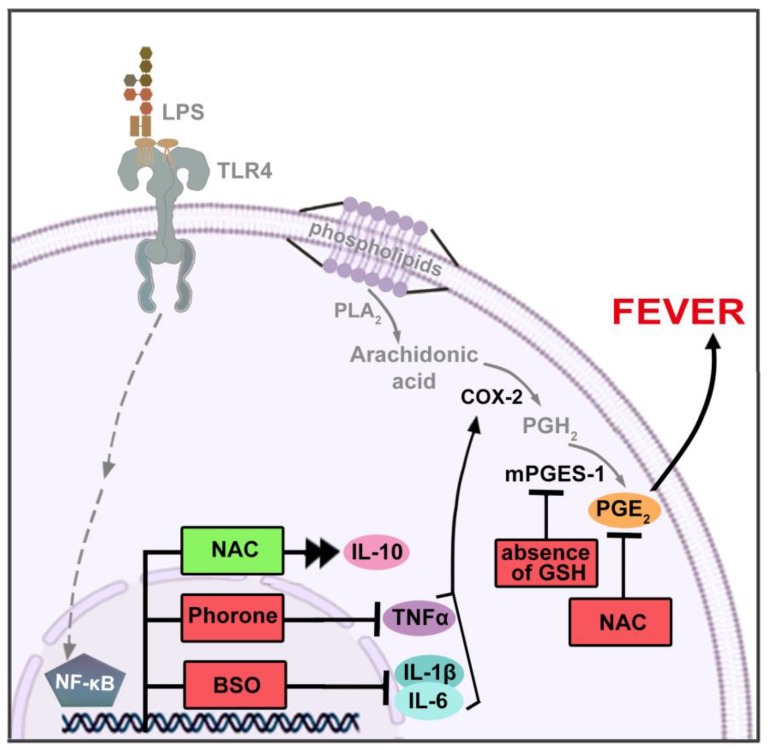
The effect of glutathione (GSH) and its modulators on the mechanism of fever. Detailed description of the fever mechanism is presented in Figure 1. The influence of glutathione and its modulators is depicted by double arrowheads and green color (stimulation) or blunt ends and red color (inhibition). *N*-acetyl-l-cysteine (NAC)—glutathione precursor; buthionine sulfoximine (BSO) and phorone—glutathione depletors.

A growing body of scientific evidence demonstrates that GSH affects the key factors involved in the mechanism of fever [84,85,86,87,88] (Figure 4). GSH affects the activation of NF-κB [87,88], which induces the expression of various pro-inflammatory genes, including those encoding cytokines. The precursors of GSH (e.g., *N*-acetyl-l-cysteine, NAC) inhibit NF-κB activation [89], and, surprisingly, depletion of this thiol down-regulates NF-κB transactivation [90]. GSH also affects the secretion of cytokines such as IL-1β, IL-6, and TNF-α, which are known to have pyrogenic properties [85]. Schmidt and co-workers [91] found a significant increase of GSH in neuronal cells after treatment with IL-6. Lou and Kaplowitz [90] also demonstrated that GSH depletion can inhibit TNF-induced NF-κB transactivation.

There is also evidence that GSH affects prostaglandin E_2_ (PGE_2_), the final mediator of fever, derived from arachidonic acid. Arachidonic acid is metabolized by enzymes such as cyclooxygenase (COX-2) and microsomal prostaglandin E_2_ synthase (mPGES-1), which belong to the MAPEG (membrane-associated proteins involved in eicosanoid and glutathione metabolism) family (Figure 4). Under basal conditions, mPGES-1 is weakly expressed in most cells, and its expression is strongly induced by inflammatory factors. mPGES-1 preferentially catalyzes PGE_2_ synthesis from COX-2, rather than COX-1, derived PGH_2_ [92]. Engblom et al. [93] observed that mPGES-1 deletion resulted in a reduction of PGE_2_ levels in the central nervous system, accompanied by no fever reaction after peripheral injection of LPS. To rule out the possibility of a defect in PGE_2_ signaling, they injected PGE_2_ and found that mPGES-1 lacking mice and wild type controls develop similar fever. Moreover, in the absence of GSH, no activity of mPGES-1 is seen [84,86], which, based on current knowledge, prevents the development of prostaglandin-dependent fever.

### 4.2. Direct Evidence of the Effect of Modulation of Glutathione Level on the Mechanism of Fever

As early as 1939, the first investigations were being conducted to determine the effects of GSH on fever [94]. The authors found that GSH injection into rabbits provoked a dose-dependent decline in fever. However, this issue was not further investigated for more than 70 years. Then, in 2010, the role of GSH in the fever process again became a subject of research. Kolesnichenko et al. [95] found that a decrease in GSH level after administration of depletors such as buthionine sulfoximine (BSO) and diethyl maleate (DEM) led to a decrease in body temperature in rats. Similarly, Costa and Murphy [96] previously reported that a GSH-conjugating reagent called DEM decreased body temperature by 2–3 degrees. This data demonstrates that alteration of redox homeostasis associated with GSH depletion affects normal body temperature.

#### 4.2.1. Fever in Rats Treated with *N*-acetyl-l-cysteine (NAC)

There is now a growing body of direct evidence showing that fever may be a GSH-sensitive mechanism [15,37,38,97]. Most of this research uses pharmacological modulations of GSH level, either by depleting GSH or increasing the amount of GSH in animals. 

As we mentioned above, cysteine is frequently identified as rate-limiting, which provides the rationale as to why *N*-acetylcysteine (NAC) is used as a supplement for GSH support [69]. NAC is a substance widely used in experimental research [98,99] and clinical studies [100,101]. Although the anti-inflammatory properties of NAC have been described in various experimental studies [102,103], its effect on fever was not previously known. Wrotek et al. [15] investigated the effect of NAC on the course of the circadian rhythm of body temperature and motor activity. It was shown that injection of NAC alone did not interfere with these activities in control rats. Next, the effect of NAC on infectious and aseptic fever was tested. These two types of febrile response, although having different etiologies, exhibit similar molecular mechanisms, as they are cytokine- and PGE_2_-dependent [104,105,106]. It was found that the administration of NAC before the induction of infectious or aseptic fever significantly inhibited both of them. These antipyretic properties of NAC were not previously known. One year later, Ferreira et al. [107] found that central administration of NAC prevented baker’s-yeast-induced fever, but did not alter the febrile response elicited by PGE_2_.

Due to the fact that the onset of fever is accompanied by *sickness behavior*, which may lead to loss of body mass, the influence of GSH on these parameters was also tested by Wrotek et al. [15]. The injection of NAC did not affect the weight loss observed in animals treated with LPS but did affect weight loss in rats treated with turpentine. These findings indicate that the mechanism leading to a decrease in body mass has a different molecular basis in the different types of fever, and only the decrease in body mass due to necrotic fever is GSH-sensitive. Furthermore, the loss of body mass accompanying endotoxic fever is not a direct consequence of the increase in body temperature, because in animals in which fever was inhibited by NAC, a decrease in body weight was still observed. Therefore, the decrease in body mass is likely to be caused by other mechanisms associated with inflammation. Additionally, NAC lessened the decrease in locomotor activity induced by pyrogens. These results showed that NAC has antipyretic properties, but the molecular mechanisms underlying this phenomenon were still unknown. Further investigation revealed that fever inhibition may be associated with the activation of endogenous antipyretics such as IL-10. 

It has been shown that pretreatment of peripheral blood mononuclear cells (PBMCs) with NAC prior to LPS stimulation results in increased IL-10 levels compared to cells treated with LPS only. Furthermore, NAC reduces PGE_2_ release by LPS-stimulated PBMCs. These results show that NAC might affect fever by regulating IL-10. Further in vivo experiments revealed that the administration of IL-10-specific antibodies to rats treated with LPS and NAC significantly abolishes the antipyretic properties of NAC. The conclusion, therefore, was that the antipyretic properties of NAC are associated with increased synthesis of antipyretic IL-10 [97]. 

Other research demonstrated that NAC is able to reduce fever induced by methamphetamine when injected before, as well as during, body temperature increase. This suggests that NAC can potentially be used in the clinic to aid in the recovery from various hyperthermic states due to drug abuse and infection [15,108] (Figure 4).

#### 4.2.2. Fever in Organisms with Pharmacologically Decreased Glutathione Level

The role of GSH in fever was also investigated using rats pretreated with GSH depletors such as phorone (2,6-dimethyl-2,5-heptadiene-4-one), a compound frequently used to reduce GSH levels [109,110]. Surprisingly, it was found that GSH-depleted rats exhibited a decreased febrile response after administration of a pyrogenic dose of endotoxin compared to animals with increased GSH levels. Additionally, these animals displayed lower levels of TNF-α in blood plasma compared to those individuals having a normal concentration of GSH in the body [38]. To verify the role of TNF-α in this observation, it was replenished by the injection of recombinant TNF-α. This procedure was able to partially restore fever, which indicated that even low concentrations of GSH in organisms can modulate endotoxic fever, and this change is TNF-α-dependent. This result is consistent with that reported by other authors who showed that the synthesis of TNF-α requires a signaling pathway in which GSH is involved [111]. In addition, these results suggest that reduced GSH levels in the body can also inhibit other factors involved in the induction of fever (Figure 4).

Besides phorone, an alternative approach to the investigation of fever that uses BSO (buthionine sulfoximine) to decrease GSH levels was proposed. The mechanism of BSO action differs significantly from that of phorone. In phorone-dependent GSH depletion, an enzyme called glutathione S-transferase is involved, and its action leads to the removal of GSH conjugates in bile. In contrast, BSO is an inhibitor of gamma-glutamylcysteine synthetase (gamma-GCS), a key enzyme involved in GSH synthesis [112,113,114]. Wrotek et al. [37] investigated whether BSO affects levels of pyrogenic cytokines such as IL-1β and IL-6. They found that BSO reduces the concentration of GSH, but it does not affect the normal course of core body temperature in rats. Above all, however, it has been shown that BSO reduces both the level and duration of fever induced by a pyrogenic dose of endotoxin. This effect is accompanied by a reduction in mRNA expression of IL-1β and IL-6 (Figure 4). The inhibitory effect of BSO on the gene expression of IL-1β was shown to start four hours after LPS injection, and three hours later, IL-1β was no longer detected in the plasma.

## 5. Conclusions and Perspectives

GSH is an antioxidant that, according to a growing body of evidence, is an important signaling molecule engaged in immunological processes [75,76,77,83]. Most papers indicate that increased GSH levels lead to a decrease in inflammation [102,103]. Additionally, other research has revealed that this effect is not limited to single inflammatory markers but can be observed in a complex reaction such as fever. Analysis of fever patterns may provide clues for the diagnosis of infectious disease. For example, the presence of low grade intermittent fever and night sweats is an informative symptom that may indicate extrapulmonary tuberculosis [6]. Therefore, it is important to recognize all the factors that may affect fever. Published data indicate that organisms supplemented with the GSH precursor NAC display weakened infectious and aseptic fevers [15]. Because NAC is widely used as a mucolytic drug for the treatment of bronchial infections, the knowledge about its new antipyretic properties should be shared with clinics. In particular, it is necessary to realize that patients taking NAC will not be feverish, which can alter the patient’s true picture of the disease. 

Although the overall antipyretic mechanism induced by NAC is not known, there are data showing the involvement of IL-10 in this process. Therefore, the manipulation of GSH levels by NAC might have a therapeutic value, since it is known that, in contrast to NAC, steroidal anti-inflammatory drugs, which also inhibit fever, do not affect IL-10 levels [115,116]. It is already known that IL-10 relieves experimental colitis [117], multiple sclerosis [118], pancreatitis [119], diabetes [120], experimental endotoxemia [121], and arthritis [122]. It is possible that NAC may be used to treat these diseases. This issue, however, needs further research.

Data generated from experiments on fever in animals with decreased GSH levels revealed a surprising effect. It was expected that if elevated levels of GSH prevent fever, then low levels would have the opposite effect. However, it was shown that lowering GSH levels using BSO, rather than exacerbating fever, also suppressed it. This observation, as in the case of NAC, should be shared with clinicians who use BSO in clinics. Many years ago, Deml and Oesterle [123] observed that elevated levels of GSH in tumor cells were responsible for chemo- and radio-resistance. It has also been shown that experimental reduction in GSH levels sensitizes tumor cells to both therapies [124,125,126]. For this reason, biomedical research on the use of BSO to reduce the concentration of GSH and sensitize the tumor to therapeutics has been initiated. The effects of this compound have been seen not only in preclinical studies, but also in clinical trials [112,127]. Since many cytostatic agents can cause neutropenia, cancer patients are at higher risk of infection. It is possible that a neutropenic BSO-treated patient will not develop fever during infection. Without fever, which is often the first signal generated in response to a pathogen, the beginning of infection can be overlooked and disease can progress quickly. It is noteworthy that similar antipyrogenic properties have been observed in organisms treated with phorone, another GSH modulator.

The results obtained using animals with pharmacologically modulated levels of GSH indicate that fever develops only when the GSH level is within the normal range (steady state). Both lowering and raising its level are mechanisms of blocking the development of fever. Similarly, Kinscherf et al. [128] showed that healthy human subjects with intermediate GSH levels have a significantly higher number of CD4+ T cells than individuals with either lower or higher intracellular GSH levels. Likewise, investigations on NF-κB activation revealed that treatment with either a GSH precursor or GSH depletor inhibits NF-κB activation [89,90]. Kabe et al. [129] observed that reactive oxygen species enhance the signal transduction pathway for NF-κB activation, but the DNA binding activity of oxidized NF-κB is significantly diminished. According to Dröge and Breitkreutz [83], the immune system may be exquisitely sensitive not only to GSH deficiency, but also to an excess of GSH. Taking into consideration the statement of Riedel and Maulik [63], who believe that fever is an integrative response of the central nervous system to oxidative stress, we hypothesize that high levels of antioxidants prevent oxidative stress which, in turn, inhibits fever. On the other hand, organisms with decreased GSH levels are deprived of protection against oxidative stress. In this case, the induction of fever (which generates free radicals) would be too harmful, so to prevent it, mechanisms leading to endogenous antipyresis are induced.

The molecular mechanism of fever is still not fully understood, and more clinical research is required. Data presented in this review suggest that permanent lack of infectious fever, which is observed in some patients, may result from disrupted GSH homeostasis. Thus, the role of GSH in the mechanism of fever has to be more clearly identified, and additional research is warranted.
